# Sex specific impact of different obesity phenotypes on the risk of incident hypertension: Tehran lipid and glucose study

**DOI:** 10.1186/s12986-019-0340-0

**Published:** 2019-02-27

**Authors:** Maryam Kabootari, Samaneh Akbarpour, Fereidoun Azizi, Farzad Hadaegh

**Affiliations:** 1 Metabolic Disorders Research Center, Golestan university of Medical Sciences, Gorgan, Iran; 2 Prevention of Metabolic Disorders Research Center, Research Institute for Endocrine Sciences, Shahid Beheshti University of Medical Sciences, P.O. Box: 19395-4763, No. 24, Parvaneh Street, Velenjak, Tehran, Iran; 3 Occupational Sleep Research Center, Baharloo Hospital, Tehran University of Medical Sciences, Tehran, Iran; 4 Endocrine Research Center, Research Institute for Endocrine Sciences, Shahid Beheshti University of Medical Sciences, Tehran, Iran

**Keywords:** Obesity phenotype, Hypertension, Insulin resistance, Metabolic syndrome

## Abstract

**Background:**

To investigate the association between different obesity phenotypes and the risk of incident hypertension among both genders.

**Methods:**

The study population included 3659 Iranians (me*n* = 1540), aged ≥20 years free of hypertension at baseline. Participant**s** were classified into six categories of body mass index (BMI)-metabolic health status, in which unhealthy metabolic status was defined based on the presence of > 1 component of metabolic syndrome (MetS) using the joint interim statement (JIS) criteria or the presence of insulin resistance (IR). The association between different obesity phenotypes and incident hypertension was assessed using multivariate Cox’s proportional hazard models adjusted for age, current smoking, low physical activity, diabetes mellitus, family history of premature cardiovascular disease, estimated glomerular filtration rate, phase of recruitment, BMI and systolic blood pressure, considering metabolically healthy normal weight group as the reference.

**Results:**

After a median follow-up of 11.6 years 1122 participants (men = 493) experienced hypertension. Using JIS criteria, a significant higher risk of hypertension was observed among metabolically healthy obese and well as metabolically unhealthy groups among men in the age adjusted model; however, a significant higher risk in the fully adjusted model was seen among women in the metabolically healthy obese [hazard ratio (HR) 95% confidence interval (CI) 1.96(1.16–3.32)] as well as metabolically unhealthy normal weight [1.98(1.37–2.86)], overweight [2.08(1.49–2.90)] and obese [2.06(1.27–3.30)] groups. Using insulin sensitive normal weight group as the reference, among men, being overweight or obese with and without IR was significant predictors of incident hypertension in the age adjusted model; however, among women, insulin resistant overweight [1.46(1.06–2.02)] and obese groups, [1.63(1.01–2.62)] showed significant risk in the fully adjusted model.

**Conclusion:**

We concluded that first, there was significant difference between genders in the associations between obesity phenotypes and incident hypertension. Second, in general, metabolic status defined by MetS components as compared to IR could do better in identifying high risk women for hypertension. Third, women populations who are metabolically healthy obese using MetS definition or those with either > 1 component of metabolic syndrome or overweight/obese ones with IR should be prioritized for implementing urgent preventive strategies against hypertension focusing on lifestyle changes.

**Electronic supplementary material:**

The online version of this article (10.1186/s12986-019-0340-0) contains supplementary material, which is available to authorized users.

## Introduction

Obesity is a serious public health concern worldwide [1]. Both general and central obesity show an increasing trend particularly during recent years among Iranian populations in the background of Westernization of diets and sedentary lifestyle [2]. Individuals with obesity are at increased risk of type 2 diabetes, hypertension, cancer and cardiovascular diseases(CVD) [3]. Similarly, hypertension is an important leading cause of CVD and all-cause mortality with population attributable fractions (PAFs) of 21.62 and 17.13% for CVD and all-cause mortality, respectively, among Iranian populations [4].

Different subtypes of obesity have been defined based on combinations of obesity and metabolic components [5]. A sub-group of metabolically healthy overweight or obese individuals, has been described; however, its benign nature regarding development of CVD and all-cause mortality events remains unclear [6, 7]. Moreover, inconsistent definitions of metabolic health and obesity are used in different studies [8]; recently, an association between different obesity phenotypes including metabolically healthy obesity and development of hypertension has been described in few studies mostly conducted in East Asia and one study in the Europe [9–13]. Furthermore, to the best of our knowledge, except for one study performed only among men [13], all studies have been conducted in pooled samples and data regarding the effect of sex on the outcome is lacking.

Considering the high prevalence and incidence of obesity [14] and hypertension among a Middle Eastern populations [15], we aimed to investigate the association between different obesity phenotypes and the development of hypertension, separately in each gender considering 2 different definitions of unhealthy metabolic status i.e., the presence of more than 1 component of metabolic syndrome (MetS) or the presence of insulin resistance (IR) [16, 17] over a long term follow-up in the population-based cohort of the Tehran Lipid and Glucose Study (TLGS).

## Methods

### Study population

The TLGS is an ongoing prospective longitudinal population-based study conducted on a representative sample of Tehran, the capital city of Iran. The main aim of the study was to investigate the risk factors and outcomes of non-communicable disease. TLGS recruitment was conducted in two phases, i.e. the first, (1999–2001) and the second (2002–2005) phases and is planned to continue for at least 20 years with follow-up exams with an approximately 3-year intervals; i.e. the third phase: 2005–2008, fourth phase: 2009–2011 and the fifth phase: 2012–2015. Study protocol has been described in more detail elsewhere [18].

For the present study, we considered the data of 5454 participants, aged ≥20 years, who had measurements of insulin level at baseline. After excluding subjects with prevalent hypertension at baseline (*n* = 1024), prevalent CVD (*n* = 126), women who were pregnant during any examination (*n* = 55) and those with body mass index (BMI) < 18.5 kg/m^2^ (*n* = 108), 4141 participants were enrolled the current study. Moreover, other exclusions were those with missing data of BMI (*n* = 105), hypertension status (*n* = 76), MetS components (*n* = 98) and other covariates including smoking, family history of premature CVD, serum creatinine level, physical activity status (*n* = 139), and those without any follow-up data (*n* = 64), leaving 3659 participants for the final analysis. The study was approved by the ethics committee of the Research Institute for Endocrine Sciences and the ethical principles of the Helsinki Declaration were followed.

### Clinical and laboratory measurements

At baseline, participants were interviewed by trained personnel using pretested questionnaires. Information on age, gender, smoking habits, medical and drug history, and family history of premature CVD were collected. Weight was measured in light clothing without shoes, using a digital scale (Seca 707, Seca Corp., Hanover, MD, USA; range 0.1–150 kg) to the nearest 0.1 kg. Height was measured in a standing position without shoes using an unstretched tape meter, while shoulders were in normal alignment. BMI was measured as weight (kg) divided by square of height (m^2^). Waist circumference (WC) was measured at the umbilical level to the nearest 0.1 cm over light clothing, using a tape meter, with no pressure on body surface. Blood pressure was taken twice after 15-min resting in sitting position, using a standardized mercury sphygmomanometer on the right arm, and mean of two measurements was considered as the subject’s blood pressure. Physical activity level was assessed with the Lipid Research Clinic (LRC) questionnaire in the first phase of the TLGS [19]; due to some inaccuracies of the LRC, it was replaced from the second phase by the Modifiable Activity Questionnaire (MAQ), a questionnaire which measures all three forms of activities including leisure time, job, and household activities in the past year. [20].

After 12 h overnight fasting a venous blood sample was drawn from all study participants. Fasting plasma glucose (FPG), 2-h post challenge plasma glucose (2 h-PCPG) with 75 g glucose, total cholesterol (TC), High density lipoprotein-cholesterol (HDL-C) and triglyceride (TG) levels were measured using methods described before. [18] Insulin level was measured using the enzyme-linked immune sorbent assay (Mercodia, Uppsala, Sweden) with inter- and intra assay coefficients of variations of 3.3 and 1.4%, respectively.

### Definition of terms

Obesity phenotype was defined based on the WHO BMI definition as follows: Normal weight BMI < 25 kg/m^2^, overweight BMI ≥ 25 and < 30 kg/m^2^ and obese BMI ≥ 30 kg/m^2^. Hypertension was defined as systolic blood pressure (SBP ≥ 140 mm-Hg) and/or diastolic blood pressure (DBP ≥ 90 mm-Hg) or under treatment with anti-hypertensive drugs. Metabolic abnormalities were defined by the presence of MetS components. MetS components for this study included the criteria proposed by the Joint Interim Statement (JIS) [16] as follows: (1) central obesity defined as WC ≥90 cm, the cut off proposed for the adult Iranian population for both sexes [21, 22]; (2) FPG ≥100 mg/dl (5.6 mmol/l) or drug treatment; (3) low serum HDL-C defined as < 50 mg/dl (1.29 mmol/l) in women and < 40 mg/dl (1.03 mmol/l) in men or drug treatment; (4) elevated blood pressure defined as SBP ≥ 130 mmHg or DBP ≥ 85 mmHg or antihypertensive drug treatment; and (5) hypertriglyceridemia, defined as fasting TG levels ≥150 mg/dl (1.7 mmol/l) or drug treatment for elevated TG level. In the present study, participants were considered as metabolically healthy if they had ≤1 components of the MetS syndrome. IR was evaluated by the hemostasis model assessment-insulin resistance (HOMA-IR) index, using the following equation: HOMA-IR = fasting insulin (μU/mL) × fasting glucose (mmol/l)/22.5; IR was defined as HOMA-IR ≥2.17 among men and HOMA-IR ≥1.85 among women [23].

Family history of premature CVD was defined as history of CVD among first degree female relatives aged < 65 years or first degree male relative aged < 55 years. Smoking status was stratified as smoker (current smokers) versus nonsmoker (including past and never smokers). Type 2 diabetes was defined as FPG ≥7 mmol/L, 2 h-PCPG ≥11.1 mmol/L or using anti-hyperglycemic drugs. Estimated glomerular filtration rate (eGFR) expressed as mL/minute/1.73 m^2^, was estimated using the abbreviated prediction equation, calculated using the CKD Epidemiology Collaboration (CKD-EPI) equation [24]. CKD was defined as eGFR of < 60 ml/min per 1.73 m^2^ [25]. Being physically active was defined as individuals participating in vigorous physical activity at least three days per week for those who entered in the first phase of the TLGS. Those participants who entered the second phase, were considered physically active when achieving a minimum of at least 600 MET (metabolic equivalent task)-minutes per week [20].

Participants were classified into six phenotypes based on BMI category and metabolic health: 1) metabolically healthy normal weight, 2) metabolically healthy overweight, 3) metabolically healthy obese, 4) metabolically unhealthy normal weight, 5) metabolically unhealthy overweight and 6) metabolically unhealthy obese. We also used IR to define metabolic abnormalities. Similarly, six phenotypes were defined according to IR and different BMI categories.

### Statistical analysis

All data are expressed as mean (SD) or median (interquartile range) for continuous variables and percentage for categorical ones. The mean difference [95% Confidence interval (CI)] of continuous variables and mean differences in the prevalence [95% CI] of each category of categorical variables were estimated to compare respondents with non-respondents i.e. missing data of covariates at the baseline or those without any follow up.

Differences of baseline characteristics between groups were analyzed using one-way ANOVA for continuous variables (Kruskal Wallis for HOMA-IR) and Chi-squared test for categorical ones. Event date was considered as the time of incident hypertension. Censoring time was defined as time point of leaving the residential area, death, loss to follow-up or end of study (19 January 2015), whichever occurred first. The event date for participants with incident hypertension was defined as mid-time between the date of the follow-up appointment at which hypertension was detected for the first time and the last follow-up visit before the diagnosis; for those with a negative event (censored subjects), the survival time was defined as the difference between the first and the last observation dates.

Cox’s proportional hazard models were used to evaluate associations between different obesity phenotypes and development of incident hypertension, considering metabolically healthy normal weight as reference groups. Furthermore, these associations were evaluated using insulin sensitive normal weight individuals as reference groups.

Associations were evaluated in 3 models: Model 1, included age; Model 2 was further adjusted for current smoking status, low physical activity, diabetes mellitus, family history of premature CVD, eGFR, phase of recruitment and SBP and model 3; model 2 plus BMI.

In the multivariate analysis, the effect modifications of gender on the associations between different obesity phenotypes and incident hypertension were tested by entering the interaction terms (obesity phenotype × gender) in the model. There were significant effect modifications of gender on the metabolically healthy normal weight (*P* = 0.001), metabolically healthy overweight (*P* = 0.04), metabolically unhealthy overweight (*P* = 0.05) and metabolically unhealthy obese (*P* = 0.04) groups. Therefore, we stratified our analysis by gender. However, to make our finding comparable with those of other studies in this field, the analysis was also performed in the pooled sample which included sex as a covariate. Statistical significance was defined as a *p*-value of less than 0.05 in a two-sided manner. Statistical analyses were conducted using Stata version 12.0 (Stata Corp LP, College Station, Texas).

## Results

A total of 3659 participants (men = 1540) were evaluated. Baseline characteristics of respondent and non-respondent groups are compared in Additional file 1: Table S1, indicating no clinically important differences between respondent and non-respondent groups. Baseline characteristics of the study population among 6 different obesity phenotypes, defined by JIS criteria of metabolic syndrome, among men and women are presented in Tables 1 and 2, respectively. The mean (SD) of age, BMI, SBP and DBP among men were 40.0(13.32) years, 25.70(3.82) kg/m^2^, 113.99(11.0) mm-Hg and 74.26(8.22) mm-Hg, respectively; corresponding values among women were 38.20(11.65) years, 26.95(4.44) kg/m^2^, 110.71(11.54) mm-Hg and 73.80(7.97) mm-Hg, respectively. Compared to metabolically healthy normal weight participants, generally, subjects with other obesity phenotypes in both sexes were older and had more unfavorable metabolic profiles, excluding similar low physical activity status among women.

**Table 1:** Baseline characteristics of study population based on combinations of body mass index and metabolic health defined by JIS criteria^a^ for men

		Metabolically healthy (*n* = 457)			Metabolically unhealthy (*n* = 1083)			
Characteristics	Total (*n* = 1540)	Normal weight (*n* = 369)	Overweight (*n* = 81)	Obese (*n* = 7)	Normal weight (*n* = 322)	Overweight (*n* = 568)	Obese (*n* = 193)	*P*-value (for trend)
Age(years)	40.0(13.32)	35.49(13.39)	38.30(12.92)	42.28(14.47)	41.22(13.95)	41.94(12.62)	41.42(12.30)	< 0.0001
BMI (kg/m^2^)	25.70(3.82)	21.91(1.73)	26.30(1.16)	32.89(4.04)	22.94(1.59)	27.34(1.36)	32.22(2.69)	< 0.0001
WC (cm)	88.55(10.60)	77.81(5.54)	86.60(4.73)	105.14(10.82)	82.35(6.52)	93.97(5.43)	103.69(7.50)	< 0.0001
SBP (mm-Hg)	113.99(11.00)	108.24(9.67)	111.55(9.31)	116.57(8.38)	115.08(11.86)	116.16(10.55)	117.70(9.62)	< 0.0001
DBP (mm-Hg)	74.26(8.22)	70.21(7.94)	71.32(7.08)	74.42(6.47)	74.35(8.67)	76.02(7.54)	77.90(6.86)	< 0.0001
Low Physical activity (%)	1452(68.5)	249(67.5)	55 (67.9)	4 (57.1)	228(70.8)	400 (70.4)	148(76.7)	0.002
Current smoker (%)	82(3.9)	127 (34.4)	24(29.6)	3(42.9)	100(31.1)	177(31.2)	50(25.9)	0.029
Family history of premature CAD (%)	334(15.4)	38(10.3)	11(13.6)	0	37(11.5)	83(14.6)	36(18.7)	< 0.0001
FPG (mmol/l)	5.25(1.37)	4.79(0.37)	4.80(0.41)	5.12(0.21)	5.32(1.28)	5.44(1.57)	5.64(1.97)	< 0.0001
Total cholesterol (mmol/l)	5.12(1.05)	4.55(0.91)	4.97(0.91)	5.65(0.95)	5.21(1.02)	5.37(1.07)	5.36(0.91)	< 0.0001
TG (mmol/l)	1.97(1.27)	1.07(0.33)	1.24(0.29)	1.18(0.22)	2.14(1.07)	2.43(1.51)	2.43(1.14)	< 0.0001
HDL-C(mmol/l)	0.97(0.23)	1.10(0.26)	1.02(0.26)	1.44(0.24)	0.94(0.18)	0.90(0.19)	0.89(0.19)	< 0.0001
eGFR (ml/min/1.73 m^2^)	76.25(11.83)	79.39(12.07)	75.39(11.04)	69.95(5.33)	76.31(11.61)	74.86(11.61)	74.86(11.80)	< 0.0001
HOMA-IR	1.92(1.66)	1.15(0.53)	1.44(0.69)	2.25(1.79)	1.88(1.11)	2.18(1.49)	2.85(1.83)	< 0.0001

Values are expressed as mean ± SD except median (interquartile range) for TG and HOMA-IR

JIS, Joint Interim Statement; BMI, body mass index; WC, waist circumference; SBP, systolic blood pressure; DBP, diastolic blood pressure; FPG, fasting plasma glucose; TG, triglyceride; HDL-C, High density lipoprotein-cholesterol; eGFR, estimated glomerular filtration rate; HOMA-IR, hemostasis model assessment-insulin resistance

^a^Participants considered as metabolically healthy if they had ≤1 of the JIS components

**Table 2:** Baseline characteristics of study population based on combinations of body mass index and metabolic health defined by JIS criteria^a^ for women

		Metabolically healthy (*n* = 1231)			Metabolically unhealthy (*n* = 888)			
Characteristics	Total (*n* = 2119)	Normal weight (*n* = 629)	Overweight (*n* = 473)	Obese (*n* = 129)	Normal weight (*n* = 130)	Overweight (*n* = 388)	Obese (*n* = 370)	*P*-value (for trend)
Age(years)	38.20(11.65)	32.08(10.43)	36.57(10.07)	40.41(10.95)	42.36(12.72)	42.67(11.01)	43.74(10.59)	< 0.0001
BMI (kg/m2)	26.95(4.44)	22.33(1.76)	27.08(1.39)	32.81(2.66)	23.34(1.37)	27.59(1.42)	33.18(2.81)	< 0.0001
WC (cm)	85.51(11.62)	74.51(6.68)	83.82(6.35)	94.17(9.39)	80.50 (7.23)	90.14(7.19)	100.24(7.70)	< 0.0001
SBP (mm-Hg)	110.71(11.54)	105.39(10.48)	108.74(9.94)	111.37(8.75)	114.41(13.63)	114.82(11.39)	116.45(10.72)	< 0.0001
DBP (mm-Hg)	73.80(7.97)	70.16(7.96)	72.60(7.28)	74.98(6.03)	75.18(8.45)	76.47(7.42)	77.80(6.67)	< 0.0001
Low Physical activity (%)	1084(70.4)	439(69.8)	302(63.8)	88(68.2)	93(71.5)	267(68.8)	263(71.1)	0.304
Current smoker (%)	481(31.2)	21(3.30)	19(4.00)	6(4.7)	4(3.1)	15(3.9)	17(4.6)	0.043
Family history of premature CAD (%)	205(13.3)	83(13.2)	66(14.00)	28(21.7)	17(13.1)	75 (19.3)	65 (17.6)	0.029
FPG (mmol/l)	5.15(1.41)	4.74(0.47)	4.82(0.62)	4.77(0.43)	5.98(2.72)	5.65(1.90)	5.57(1.72)	< 0.0001
Total cholesterol (mmol/l)	5.20(1.13)	4.70(0.94)	5.10(1.02)	5.32(0.88)	5.42(1.10)	5.59(1.21)	5.65(1.18)	< 0.0001
TG (mmol/l)	1.60(0.99)	1.04(0.47)	1.22(0.60)	1.21(0.36)	2.22(1.02)	2.30(1.12)	2.19(1.14)	< 0.0001
HDL- C(mmol/l)	1.15(0.28)	1.24(0.27)	1.23(0.25)	1.32(0.25)	1.00(0.26)	0.99(0.25)	1.05(0.26)	< 0.0001
eGFR (ml/min/1.73 m2)	70.93(11.11)	75.81(10.99)	70.47(9.95)	69.03(11.34)	68.96(10.56)	68.15(10.50)	67.51(10.63)	< 0.0001
HOMA-IR	2.06(1.63)	1.52(0.72)	1.74(0.90)	1.98(1.10)	2.45(3.66)	2.49(1.75)	2.84(1.89)	< 0.0001

Values are expressed as mean ± SD except median (interquartile range) for TG and HOMA-IR

JIS, Joint Interim Statement; BMI, body mass index; WC, waist circumference; SBP, systolic blood pressure; DBP, diastolic blood pressure; FPG, fasting plasma glucose; TG, triglyceride; HDL-C, High density lipoprotein-cholesterol; eGFR, estimated glomerular filtration rate; HOMA-IR, hemostasis model assessment-insulin resistance

^a^Participants considered as metabolically healthy if they had ≤1 of the JIS components

During a median follow-up of 11.65 (interquartile range, 6.5) years, hypertension was observed in 493 men and 629 women (30.7% of total population); corresponding incidence rates were 32.09 and 28.09 per 1000 person- years, respectively.

Table 3 presents the risk for hypertension among different obesity phenotypes defined by JIS criteria and IR among men and women. Considering JIS criteria to define metabolic health among men and women, metabolically healthy obesity as well as the metabolically unhealthy normal weight, overweight and obese groups were associated with greater risk of hypertension among men in the age adjusted model; moreover, metabolically unhealthy obese group showed a 63% greater risk considering covariates in model 2; however, these risks reached to null after further adjustment for BMI. In contrast, among women metabolically healthy obese, metabolically unhealthy normal weight, overweight and obese groups were associated with significant higher risk of hypertension even in the fully adjusted model [HR (95%CI) of 1.96(1.16–3.32), 1.98(1.37–2.86), 2.08(1.49–2.90) and 2.06 (1.27–3.30), respectively].

**Table 3:** Risks for hypertension based on BMI and metabolic status as defined by MetS components or insulin resistance among men and women: Tehran lipid and Glucose study

	N (event)	Model 1^a^		Model 2^b^		Model 3^c^	
		HR	95% CI	HR	95% CI	HR	95% CI
Men							
Defined by MetS components^c^							
Metabolically healthy normal weight	369(66)	1.00		1.00		1.00	
Metabolically healthy overweight	81(19)	1.37	0.82–2.28	1.19	0.71–1.99	0.98	0.58–1.71
Metabolically healthy obese	7(3)	3.20	1.00–10.21	2.31	0.72–7.44	1.53	0.44–5.29
Metabolically unhealthy normal weight	322 (98)	1.50	1.09–2.05	1.07	0.78–1.48	1.03	0.75–1.43
Metabolically unhealthy overweight	568(216)	2.05	1.55–2.71	1.32	0.98–1.76	1.05	0.72–1.52
Metabolically unhealthy obese	193(91)	2.65	1.93–3.64	1.63	1.16–2.27	1.06	0.61–1.83
Defined by insulin resistance^d^							
Insulin sensitive normal weight	612(144)	1.00		1.00		1.00	
Insulin sensitive overweight	426(145)	1.49	1.18–1.87	1.21	0.96–1.53	0.98	0.73–1.34
Insulin sensitive obese	84(38)	1.88	1.31–2.69	1.46	1.01–2.09	0.95	0.54–1.66
Normal weight with insulin resistance	79(20)	1.27	0.79–2.03	0.88	0.55–1.43	0.85	0.52–1.37
Overweight with insulin resistance	223(90)	1.88	1.44–2.44	1.26	0.95–1.66	0.99	0.69–1.42
Obese with insulin resistance	116(56)	2.44	1.79–3.33	1.62	1.17–2.24	1.04	0.60–1.78
Women							
Defined by MetS components^c^							
Metabolically healthy normal weight	629(75)	1.00		1.00		1.00	
Metabolically healthy overweight	473(81)	1.19	0.86–1.63	1.08	0.79–1.48	0.99	0.69–1.41
Metabolically healthy obese	129(51)	2.72	1.89–3.89	2.42	1.69–3.48	1.96	1.16–3.32
Metabolically unhealthy normal weight	130(54)	2.73	1.91–3.90	2.02	1.40–2.91	1.98	1.37–2.86
Metabolically unhealthy overweight	388(168)	3.14	2.38–4.15	2.29	1.72–3.04	2.08	1.49–2.90
Metabolically unhealthy obese	370(200)	3.81	2.9–5.01	2.55	1.92–3.38	2.06	1.27–3.30
Defined by insulin resistance							
Insulin sensitive normal weight	522(86)	1.00		1.00		1.00	
Insulin sensitive overweight	444(104)	1.19	0.89–1.58	1.09	0.82–1.46	0.99	0.71–1.37
Insulin sensitive obese	183(85)	2.17	1.61–2.94	1.79	1.32–2.43	1.42	0.89–2.27
Normal weight with insulin resistance	237(43)	1.29	0.89–1.86	1.15	0.79–1.67	1.14	0.79–1.65
Overweight with insulin resistance	417(145)	2.11	1.61–2.76	1.63	1.24–2.14	1.46	1.06–2.02
Obese with insulin resistance	316(166)	3.04	2.34–3.96	2.09	1.61–2.74	1.63	1.01–2.62

*MetS*, metabolic syndrome; *BMI*, body mass index; *N*, number; *HR*, hazard ratio; *CI*, confidence interval

^a^Model 1, adjusted for age; ^b^Model 2, adjusted for model 1 plus current smoking status, low physical activity, diabetes mellitus, family history of premature CVD, SBP, Phase of recruitment and eGFR; ^c^Model 3, adjusted for model 2 plus BMI ^d^Participants considered as metabolically healthy if they had ≤ 1 of the JIS components. ^d^Insulin resistance was defined as a HOMA-IR ≥ 2.17 among men and HOMA-IR≥ 1.85 among women [23].

Using IR to define the unhealthy metabolic status, higher risk of hypertension was seen among overweight and obese men with and without insulin resistance in the age adjusted model; furthermore, being obese with or without insulin resistance was associated with 46 and 62% higher risk for development of hypertension after adjustment with covariates in model 2 respectively, while all risks reached to null after further adjustment for BMI. Focusing on the women population, the adjusted HRs (95% CI) for incident hypertension were 1.79(1.32–2.43) for insulin sensitive obese, 1.63(1.24–2.14) for insulin resistant overweight and 2.09(1.61–2.74) for insulin resistant obese groups, respectively considering covariates in model 2. These risks remained statistically significant in the insulin resistant overweight and obese groups in the fully adjusted model [HRs (95% CI), 1.46(1.06–2.02) and 1.63(1.01–2.62), respectively].

As shown in Additional file 2: Table S2, generally the associations between obesity phenotypes with incident hypertension using JIS criteria for definitions of unhealthy metabolic status among the pooled sample using sex as another covariate were similar to those of the female population. However, focusing on IR in the pooled sample insulin sensitive obese, insulin resistant overweight and insulin resistant obese groups showed a significantly higher risk of 63, 42 and 90% after controlling for covariates in model 2, respectively. However, these risks attenuated and became insignificant after further adjustment for BMI (model 3).

## Discussion

In this cohort study during over a decade long follow-up, for the first time, we examined the association of BMI and metabolic health status with incident hypertension separately among men and women, using different definitions of unhealthy metabolic status. We showed that considering JIS criteria to define metabolic health, only among women, the metabolically healthy obesity as well as the metabolically unhealthy groups were associated with > 95% greater risk for incident hypertension in the presence of important confounders, i.e. current smoking status, low physical activity, diabetes mellitus, family history of premature CVD, eGFR, phase of recruitment, SBP and BMI. Furthermore, considering the presence of insulin resistance for defining unhealthy status, only women in the insulin resistant overweight and obese groups showed significant and independent risk of hypertension.

The association between different obesity phenotypes and incident hypertension has been described in some previous studies, mostly conducted in East Asia [9–13]; nevertheless, direct comparison of our results with those of theirs might be difficult since first, there is no standard definition for metabolically healthy status; besides, different criteria and numbers of risk factors were applied to define metabolic health; moreover, confounders were not similar in their multivariable adjusted models; second, the differences in durations of follow-up between studies is another important factor affecting the study results. Third, we did not find any study that compared the impact of different BMI-metabolic status for incident hypertension in both genders in a cohort; in fact, all of the studies in this field were performed in a pooled sample.

Our study findings regarding the association between different obesity phenotypes defined by MetS components and consequent hypertension among the pooled sample were generally in line with those of other sex-adjusted prospective studies; the first study in this field conducted among Korean adults [11], concluded that the metabolically healthy obesity (i.e. obese population without any MetS components of ATP III criteria), was associated with a higher risk compared with the reference group (metabolically healthy normal weight). Moreover, another study of Chinese adults [9] concluded that the metabolically healthy overweight/obese groups (i.e., the absence of metabolic syndrome using International diabetes federation (IDF) criteria) were associated with greater risk of hypertension. Similarly, our study indicated that among the pooled sample, all obesity phenotypes were associated with higher risk of hypertension in the fully adjusted model, except for the metabolically healthy overweight group.

In another study conducted only among Korean men [13], metabolically healthy overweight and obese phenotypes as well as other unhealthy obesity phenotypes (according to the Wildman criteria) were associated with higher risk of hypertension. Authors concluded that both metabolic health status and being overweight or obese were independently associated with the risk of hypertension. In the current study among men, metabolically healthy obese as well as other unhealthy BMI categories were at higher risk of hypertension only before adjustment for potential confounding factors and these risks reached to null in the presence of wide set of covariates including baseline BMI.

To the best of our knowledge, two studies have examined the impact of combinations of BMI-metabolic health status on incident hypertension, using insulin resistance to define unhealthy metabolic status among adults without sex stratification [10, 12]. One study conducted in Europe [12] on a population including middle-aged and elderly individuals showed that among normal weight subjects, being insulin resistant was not associated with development or progression of hypertension; whereas, being overweight and obese with and without insulin resistance increases the risk of hypertension and its progression. Hence, being overweight or obese might be harmful per se. In another study [10] using insulin resistance to define metabolic syndrome, metabolically healthy obese, metabolically unhealthy obese or non-obese groups were associated with higher risk of hypertension in the fully adjusted model, considering that researchers did not stratify the non-obese population into normal weight and overweight groups. In the current study, in the pooled sample insulin sensitive obese as well as insulin resistant overweight and obese groups showed a significant risk of 63, 42 and 90% for CVD in the confounder adjusted model (model 2); however these risk were significantly attenuated after further adjustment for BMI.

Regarding the sex specific impact of IR on incident hypertension we have previously shown that among an Iranian population, after adjusting for potential confounders only women in the highest quartile of HOMA-IR had a significantly higher incidence of hypertension, compared with those in the lowest quartile [HR: 1.80 (95% CI 1.31–2.40] [26]. In line with these results, the present study showed that only women, with insulin resistant overweight and obese phenotypes had a significant higher risk of hypertension in the fully adjusted model.

This higher impact of IR on hypertension risk among women compared to men, was recently addressed in a meta-analysis conducted by Feng Wangs et al. [27], showing an independent association between fasting insulin as well as IR with a more pronounced risk of hypertension, in women (relative risk (RR) 2.07; 95% CI 1.19–3.60) than in men (RR 1.48; 95% CI 1.17–1.88). Likewise, the study conducted by Petrie et al. [28] showed that in women (but not men) low insulin sensitivity, measured by the euglycemic clamp technique, was associated with higher levels of SBP over time, independent of key covariates including age, baseline blood pressure, BMI and change in BMI. The higher impact of IR in the development of hypertension among women, might be attributable to the effect of lower lean body mass in women; hence, fasting insulin or HOMA-IR values in women reflect a higher level of tissue IR [28]. On the other hand, other studies showed similar or higher impact of IR among men rather than women [29, 30]. Considering the above findings, additional studies are required to investigate the mechanism for the difference in the association between IR and hypertension between genders.

Several strengths of this study include a large sample size with a long-term follow-up duration, adequate adjustment for potential confounders, reliable measurements of different covariates and last but not least a population sample representative of the Iranian population. We also examined the association between BMI and metabolic health status with incident hypertension for the first time among men and women separately using different definitions of unhealthy metabolic status. Of the limitations that should be noted, first, we did not assess changes of BMI and metabolic profiles during follow-up. Second, we did not evaluate some risk factors of hypertension, including family history of hypertension and dietary salt intake. Third, the statistically insignificant finding among metabolically healthy obese men group should be interpreted with caution due to the limited number of individuals and events, possibly leading to an unstable estimation. Fourth, as this study was performed in an Iranian population, its generalizability to other ethnicities might not be applicable.

In conclusion, our findings among an Iranian population showed that there were significant differences between genders in the association between obesity phenotypes and incident hypertension using different definitions. Generally, using JIS criteria, metabolically healthy obesity as well as all unhealthy obesity phenotypes were associated with about 2 fold higher risk of hypertension among women. Similarly, only among women, the presence of IR is a key promoter for the development of hypertension among those with BMI > 25 kg/m^2^. Moreover, in general, metabolic status defined by MetS components as compared to IR could do better in identifying high risk women for hypertension. Hence, women populations who are metabolically healthy obese using MetS definition or those with either > 1 component of metabolic syndrome or overweight/obese ones with HOMA-IR ≥ 1.85 should be prioritized for implementing urgent preventive strategies against hypertension focusing on lifestyle changes.

## Additional files

Additional file 1: **Table S1.** Comparison of baseline characteristics between respondent and non-respondent groups: Tehran Lipid and Glucose Study. (DOCX 17 kb)

Additional file 2: **Table S2.** Risk for hypertension based on BMI and metabolic status as defined by MetS components or insulin resistance among the pooled sample: Tehran lipid and Glucose study. (DOCX 18 kb)

## Abbreviations

2 h-PCPG: 2-h post challenge plasma glucose
BMI: Body mass index
CAD: Coronary artery disease
CI: Confidence interval
CKD-EPI: Chronic kidney disease Epidemiology Collaboration
CVD: Cardiovascular diseases
DBP: Diastolic blood pressure
eGFR: Estimated glomerular filtration rate
FPG: Fasting plasma glucose
HDL-C: High density lipoprotein-cholesterol
HOMA-IR: Hemostasis model assessment-insulin resistance
HR: Hazard ratio
IDF: International diabetes federation
IR: Insulin resistance
JIS: Joint Interim Statement
LRC: Lipid Research Clinic
MAQ: Modifiable Activity Questionnaire
MET: Metabolic equivalent task
MetS: Metabolic syndrome
PAFs: Population attributable fractions
SBP: Systolic blood pressure
TC: Total cholesterol
TG: Triglyceride
TLGS: Tehran Lipid and Glucose Study
WC: Waist circumference
