# Supplementary material for: Sex specific impact of different obesity phenotypes on the risk of incident hypertension: Tehran lipid and glucose study
Source: Nutr Metab (Lond). 2019 Feb 27;16:16. doi: 10.1186/s12986-019-0340-0 (PMC6391753; doi:10.1186/s12986-019-0340-0)
Supplement: Supplementary file 1 — Table S1. Comparison of baseline characteristics between respondent and non-respondent groups: Tehran Lipid and Glucose Study. (DOCX 17 kb) [file 12986_2019_340_MOESM1_ESM.docx]

Supplementary table 1. Comparison of baseline characteristics between respondent and non-respondent groups: Tehran Lipid and Glucose Study.

| Characteristics | Non-respondents  (n=482) | Respondents  (n=3659) | Mean/ Proportion difference [95% CI)] |
| --- | --- | --- | --- |
| Age (years) | 37.51(13.06) | 38.95(12.41) | 1.44(0.25 to 2.63) |
| BMI (kg/m^2^) | 26.49(4.58) | 26.42(4.23) | -0.06(-0.54 to 0.41) |
| WC (cm) | 86.32(11.01) | 86.79(11.30) | 0.56(0.02 to 1.10) |
| SBP (mmHg) | 109.58(11.69) | 112.09(11.43) | 2.50(1.32 to 3.68) |
| DBP (mmHg) | 71.08(8.46) | 73.99(8.08) | 2.91(2.07 to 3.74) |
| Low Physical activity (%) | 121 (25.1) | 2536(69.3) | -0.44(-0.48 to-0.39) |
| Current smoker (%) | 65(13.5) | 563(15.4) | -0.02(-0.05 to 0.01) |
| Family history of premature CVD (%) | 50(10.4) | 539(14.7) | -0.04(-0.06 to -0.01) |
| FPG (mmol/l) | 5.02(1.13) | 5.19(1.39) | 0.17(0.04 to 0.30) |
| Total cholesterol (mmol/l) | 4.94(1.08) | 5.17(1.10) | 0.22(0.12 to 0.32) |
| TG (mmol/l) | 1.72(1.27) | 1.76(1.13) | -0.03(-0.14 to 0.07) |
| HDL-C (mmol/l) | 1.10(0.31) | 1.07(0.28) | 0.02(-0.01 to 0.05) |
| eGFR (ml/min/1.73 m^2^) | 77.70(13.19) | 73.17(11.71) | 4.52(3.39 to 5.65) |
| HOMA-IR | 1.99(2.13) | 1.83(1.18) | 0.16(-0.17 to 0.50) |

Values are expressed as mean ± SD.

BMI, body mass index; WC, waist circumference; SBP, systolic blood pressure; DBP, diastolic blood pressure; FPG, fasting plasma glucose; TG, triglyceride; HDL-C, High density lipoprotein-cholesterol; eGFR, estimated glomerular filtration rate; HOMA-IR, hemostasis model assessment-insulin resistance.
