# Supplementary material for: Sex specific impact of different obesity phenotypes on the risk of incident hypertension: Tehran lipid and glucose study
Source: Nutr Metab (Lond). 2019 Feb 27;16:16. doi: 10.1186/s12986-019-0340-0 (PMC6391753; doi:10.1186/s12986-019-0340-0)
Supplement: Supplementary file 2 — Table S2. Risk for hypertension based on BMI and metabolic status as defined by MetS components or insulin resistance among the pooled sample: Tehran lipid and Glucose study. (DOCX 18 kb) [file 12986_2019_340_MOESM2_ESM.docx]

Supplementary table 2. Risk for hypertension based on BMI and metabolic status as defined by MetS components or insulin resistance among the pooled sample: Tehran lipid and Glucose study.

|  | N (event) | Model 1^a^ | | Model 2^b^ | | | Model 3^c^ | |
| --- | --- | --- | --- | --- | --- | --- | --- | --- |
|  |  | HR | 95% CI | HR | | 95% CI | HR | 95% CI |
| Defined by MetS components^c^ | | | | | | | | |
| Metabolically healthy normal weight | 998(141) | 1.00 |  | | 1.00 |  | 1.00 |  |
| Metabolically healthy overweight | 554(100) | 1.11 | 0.86-1.44 | | 0.99 | 0.76-1.28 | 0.88 | 0.66-1.18 |
| Metabolically healthy obese | 136(54) | 2.43 | 1.77-3.34 | | 2.09 | 1.52-2.88 | 1.61 | 1.04-2.48 |
| Metabolically unhealthy normal weight | 452(152) | 2.01 | 1.58-2.54 | | 1.46 | 1.15-1.86 | 1.43 | 1.12-1.82 |
| Metabolically unhealthy overweight | 956(384) | 2.62 | 2.15-3.19 | | 1.77 | 1.44-2.17 | 1.55 | 1.21-1.99 |
| Metabolically unhealthy obese | 563(291) | 3.33 | 2.71-4.09 | | 2.14 | 1.73-2.64 | 1.64 | 1.14-2.35 |
| Defined by insulin resistance^d^ | | | | | | | | |
| Insulin sensitive normal weight | 1134(230) | 1.00 |  | | 1.00 |  | 1.00 |  |
| Insulin sensitive overweight | 870(249) | 1.35 | 1.12-1.61 | | 1.14 | 0.95-1.37 | 1.00 | 0.80-1.24 |
| Insulin sensitive obese | 267 (123) | 2.11 | 1.68-2.63 | | 1.63 | 1.30-2.05 | 1.22 | 0.86-1.73 |
| Normal weight with insulin resistance | 316(63) | 1.28 | 0.96-1.69 | | 1.01 | 0.75-1.33 | 0.98 | 0.74-1.31 |
| Overweight with insulin resistance | 640 (235) | 2.01 | 1.67-2.41 | | 1.42 | 1.17-1.71 | 1.22 | 0.97-1.54 |
| Obese with insulin resistance | 432(222) | 2.88 | 2.38-3.48 | | 1.90 | 1.56-2.31 | 1.38 | 0.97-1.96 |

MetS, metabolic syndrome; BMI, body mass index; N, number; HR, hazard ratio; CI, confidence interval.

^a^Model 1, adjusted for age, sex. ^b^Model 2, adjusted for model 1 plus current smoking status, low physical activity, diabetes mellitus, family history of premature CVD, SBP, Phase of recruitment and eGFR, ^c^ Model 3, adjusted for model 2 plus BMI. ^c^Participants considered as metabolically healthy if they had ≤ 1 of the JIS components. ^d^Insulin resistance was defined as a HOMA-IR ≥ 2.17 among men and HOMA-IR≥ 1.85 among women[[23](#_ENREF_23)].
